# An Unexpected Diagnosis After a Viral Infection: A Case of a Giant Mediastinal Ganglioneuroma

**DOI:** 10.7759/cureus.102430

**Published:** 2026-01-27

**Authors:** André Veloso, Ana Fernandes, Rute Martins, João Santos, Vanda Areias

**Affiliations:** 1 Pulmonology, Unidade Local de Saúde do Algarve, Hospital de Faro, Faro, PRT; 2 Faculty of Medicine and Biomedical Sciences, University of Algarve, Faro, PRT; 3 Radiology, Unidade Local de Saúde do Algarve, Hospital de Faro, Faro, PRT; 4 Pathology and Laboratory Medicine, Unidade Local de Saúde de Santa Maria, Lisbon, PRT

**Keywords:** endoscopic ultrasound (eus), ganglioneuroma, posterior mediastinal mass, spectral ct, thoracic mri

## Abstract

Ganglioneuromas (GN) are rare, benign tumors that develop from sympathetic ganglia. They usually appear in children and young adults and are mostly found in the posterior mediastinum or retroperitoneum. When they occur in older adults, diagnosis can be complicated due to their slow growth, vague symptoms, and similarities in imaging to other malignant masses in the mediastinum. We describe the case of a 61-year-old woman who had a persistent dry cough, wheezing, and shortness of breath after an influenza infection. Initial medical treatment was ineffective, prompting imaging that showed a heterogeneous mass in the posterior mediastinum with cystic areas and calcifications. A magnetic resonance imaging (MRI) scan revealed a well-defined lesion that was hyperintense on T2 imaging but did not invade nearby structures. Further tests, including bronchoscopy, spectral computed tomography (CT), and endoscopic ultrasound, confirmed a mostly cystic mass that compressed the esophagus but showed no infiltration. A fine-needle aspiration did not provide a diagnosis, so surgical removal was performed. The histopathological examination confirmed it was a GN. This case highlights the diagnostic difficulties of mediastinal GN in older adults, the importance of using multiple imaging methods, and the generally positive outcomes after complete surgical removal.

## Introduction

Ganglioneuromas (GN) are rare, benign neurogenic tumors arising from sympathetic ganglia, mostly found in the posterior mediastinum (41.5%), retroperitoneum (37.5%), neck (8%), or adrenal glands (21%) [1,2]. They typically occur in adolescents and young adults [3]. Despite their benign histology, GN can attain considerable size and cause compressive effects on surrounding structures, leading to symptoms such as cough, dyspnea, chest pain, and complications such as recurrent respiratory infections and pulmonary atelectasis [4-6]. These tumors are relevant because they may present initially with respiratory complaints or be incidentally discovered. Their radiological appearance can mimic primary pulmonary neoplasms or other mediastinal tumors, creating diagnostic uncertainty. The diagnostic approach generally includes chest radiography and computed tomography (CT), followed by magnetic resonance imaging (MRI) for further characterization [7,8]. However, definitive diagnosis requires histopathological analysis.

Although the tumors are benign, surgical resection is the treatment of choice, both to alleviate compressive symptoms and to exclude malignancy [1,2,4]. We present the case of a 61-year-old female with a giant mass occupying the left thoracic cavity, ultimately diagnosed as a GN through histopathological examination.

## Case presentation

**Figure 1:**
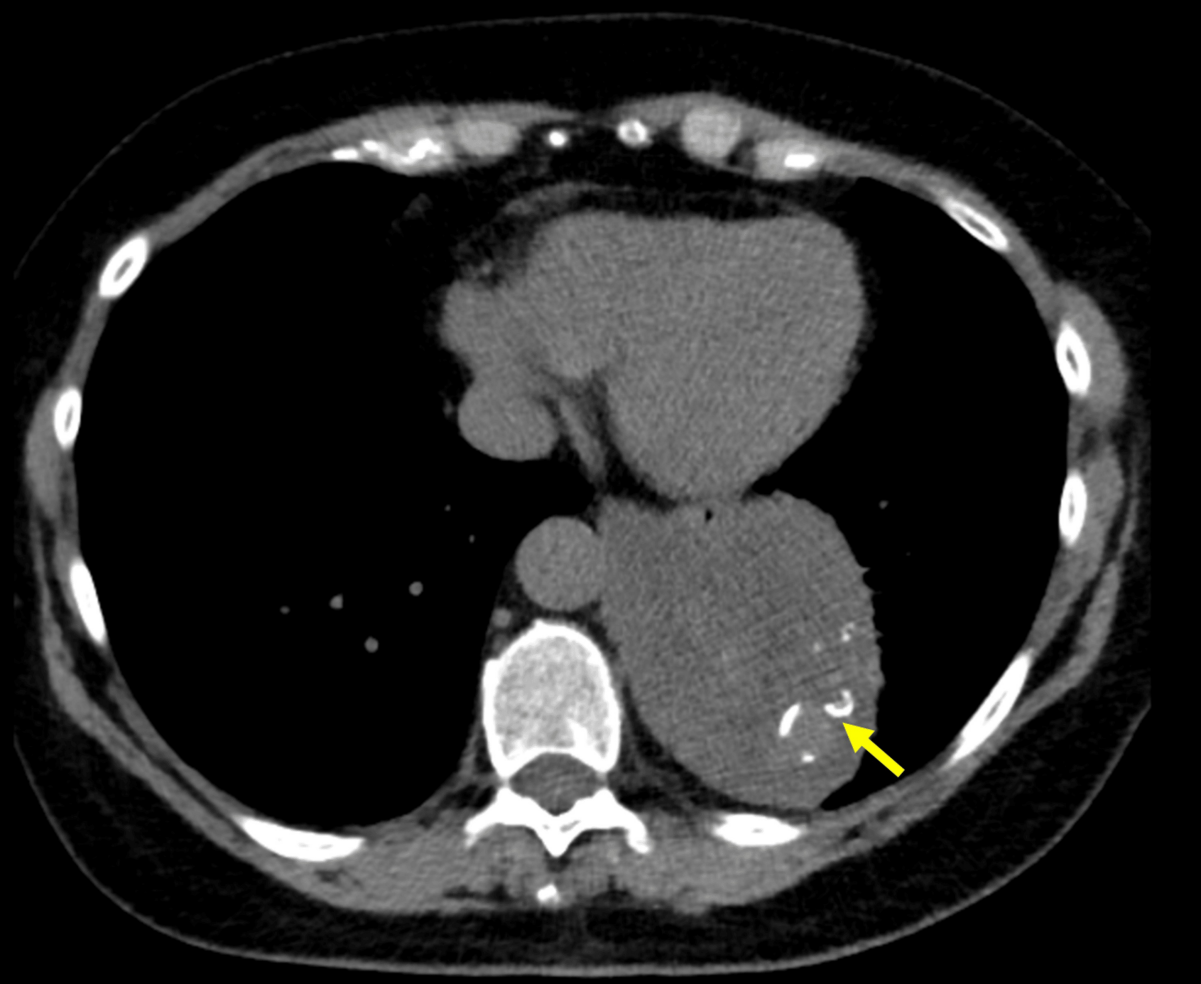
Axial thoracic CT image identifying a mass at the base of the left lung, confirming its posterior location adjacent to the vertebral column and the descending aorta (displaced anteriorly). The mass appears solid and heterogeneous, with possible cystic components and internal calcifications (yellow arrow). CT: computed tomography

We present the case of a 61-year-old Caucasian woman, a nonsmoker, with a past medical history of anxiety-depressive disorder, fibromyalgia, and dyslipidemia. The patient’s regular medications included trazodone and escitalopram. No new medications had been recently initiated. There was no history of drug allergies. She was referred for a pulmonology consultation due to post-influenza A bronchitis of three-month duration. She also reported exertional fatigue, chest tightness, and a sensation of incomplete inspiration. Additional symptoms included posterior rhinorrhea without nasal congestion or sneezing, excessive sweating, and reduced appetite in recent days. She denied hemoptysis, pyrosis, weight loss, or fever. Previous treatments with antibiotics (amoxicillin-clavulanic acid and levofloxacin), corticosteroids (prednisolone), and inhaled therapy (fluticasone furoate, umeclidinium bromide, and vilanterol) had not resulted in clinical improvement. On physical examination, the only abnormality found was decreased breath sounds at the left lung base. A non-contrast chest CT scan was performed, and a heterogeneous thoracic mass was identified at the base of the left lung with internal calcifications (Figure 1).

**Figure 2:**
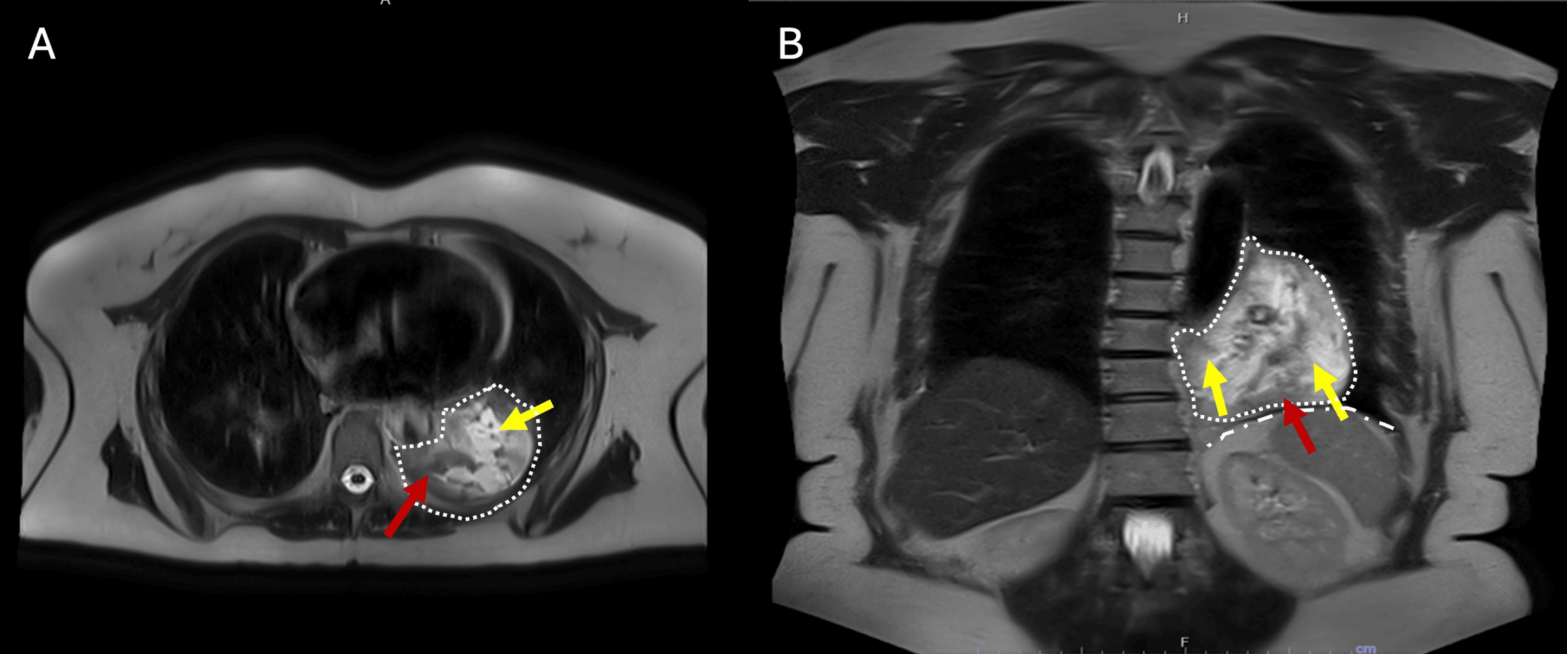
Chest MRI demonstrating the large posterior paramediastinal mass (dotted line in both images). A: Axial T2-weighted image (superior level) demonstrating the mass posterior to the heart and adjacent to the vertebral column, showing mixed high signal intensity. Some solid components were observed within the lesion without significant contrast uptake. No fat content was depicted. B: Coronal T2-weighted image showing the mass primarily located in the lower posterior mediastinum and left hemithorax, appearing markedly hyperintense and heterogeneous, consistent with its solid (red arrow) and likely partially cystic/necrotic nature (yellow arrow). The mass is seen superior to the left diaphragm (dash-dot line). MRI: magnetic resonance imaging

The patient subsequently underwent a chest MRI that, on axial T2 sequence, revealed a lesion in the left lower paravertebral region with well-defined margins, a 13.0-cm longitudinal axis, and a 9.2x6.5-cm major axis in the axial plane. The lesion showed hyperintensity on T2 and hypointensity on T1, and mild enhancement after contrast administration, without evidence of invasion of adjacent structures (Figure 2).

**Figure 3:**
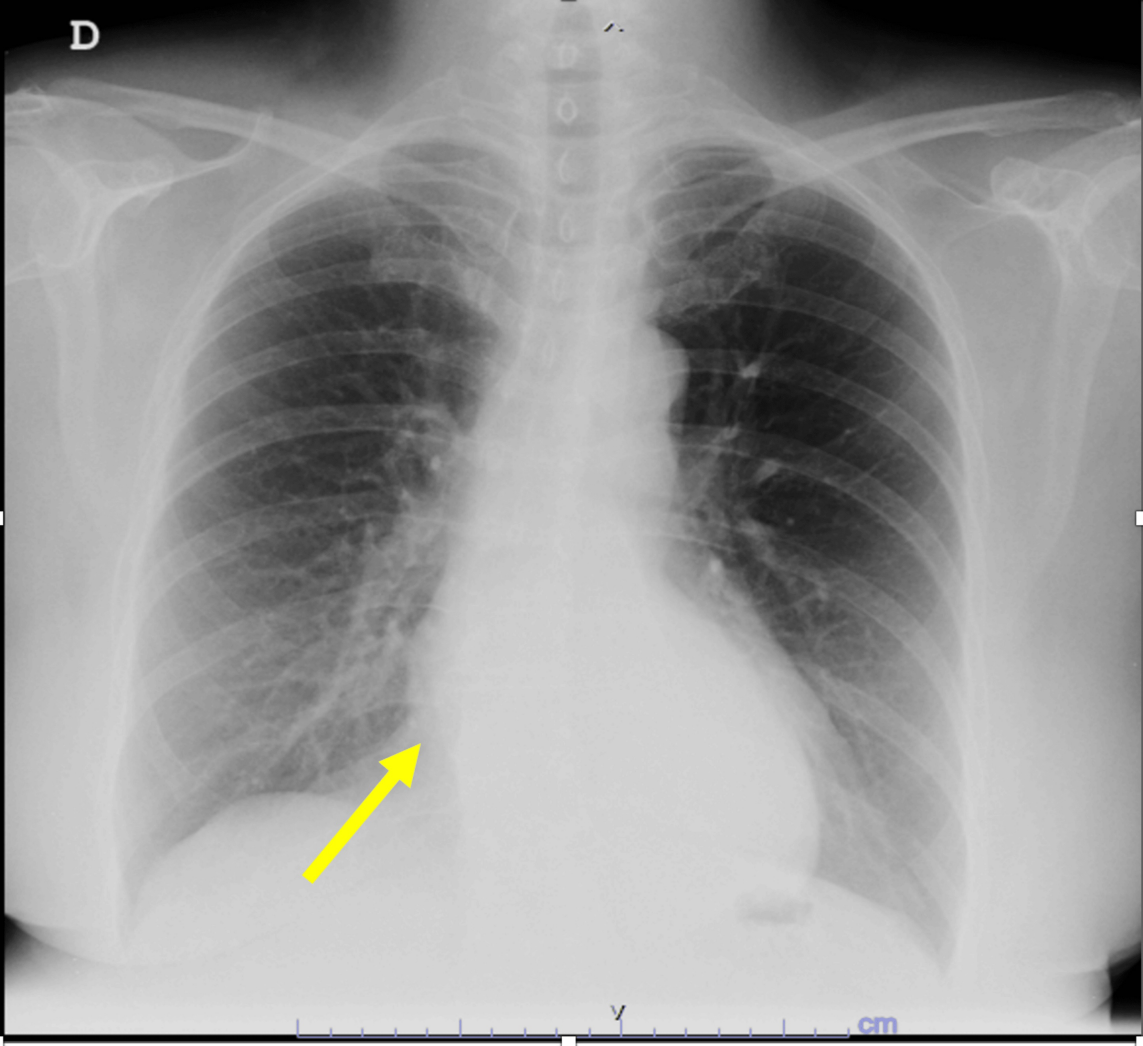
Previous chest radiograph, 14 years before the clinical presentation, demonstrates preserved lung volumes with no overt focal pulmonary consolidation or pleural effusion. The cardiac silhouette appears within normal limits in size. However, there is a subtle area of increased opacity projected over the mid-to-lower posterior mediastinum. This finding is partially obscured by the thoracic vertebral column and overlaps the right cardiac border, which may hinder precise characterization. The contour or silhouette of the suspected lesion remains medial to the right cardiac silhouette (arrow), raising the possibility of a posterior mediastinal abnormality.

Review of earlier chest radiographs (14 years before) and abdominal CT images (four years before) showed changes suspicious for a posterior mediastinal mass (Figure 3).

**Figure 4:**
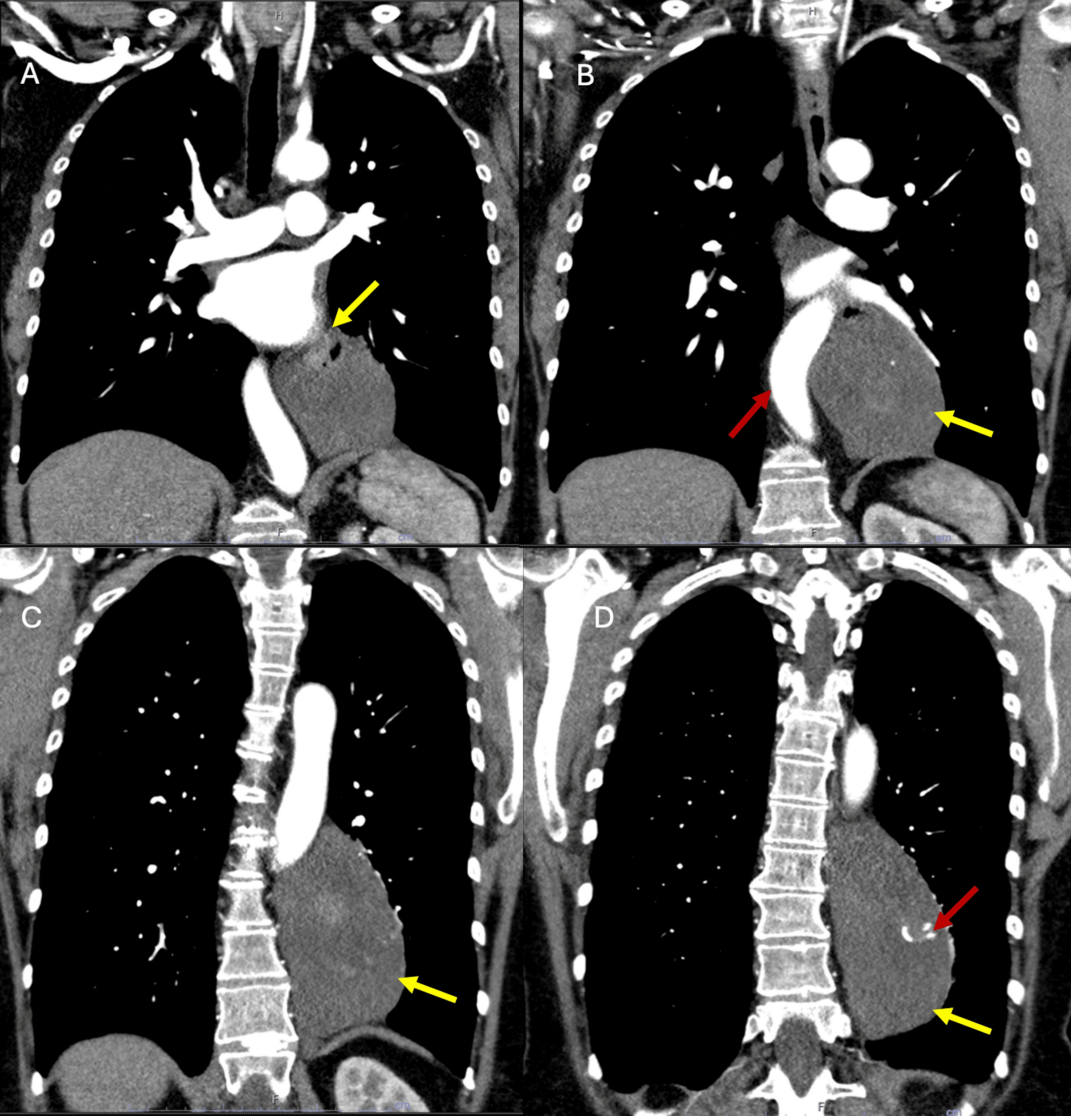
Spectral chest CT images (A-D) demonstrating a large posterior paramediastinal mass. A: More superior coronal slice demonstrating the relationship of the upper portion of the mass (yellow arrow) with the mediastinal vascular structures, specifically the great vessels and the pulmonary artery (enhanced by intravenous contrast). B: A slightly more anterior coronal slice highlighting the relationship of the mass (yellow arrow) with the descending aorta (red arrow), which appears to be displaced anteriorly. The heterogeneous density of the mass is observed. C: Mass (yellow arrow) adjacent to the descending aorta and the vertebral column, presenting a solid and heterogeneous appearance, measuring 13.0 cm longitudinally and 9.2x6.5 cm transversely. D: Posterior coronal slice demonstrating the craniocaudal extent of the mass (yellow arrow) and its close relationship with the posterior mediastinum. Note the presence of coarse calcifications (red arrow) within the lesion with a 7-mm hyperenhancing focus. CT: computed tomography

Pulmonary function tests revealed a mild restrictive ventilatory defect (total lung capacity (TLC) 3.63 L, 74% predicted, z-score -2.29). Laboratory investigations were unremarkable. Multidisciplinary discussion with radiology and thoracic surgery recommended further evaluation with bronchoscopy, endoscopic ultrasound (EUS), thoracic ultrasound, and spectral chest CT to better characterize the lesion and plan potential surgical intervention. Spectral chest CT showed a large, well-defined posterior mediastinal lesion adjacent to the vertebral column and posterior costal pleura, confirming MRI findings that the lesion caused mild compression of the esophagus but with no definite signs of invasion of the esophagus, thoracic aorta, diaphragm, or subpleural fat. There was no relationship with the vertebral column or spinal canal (Figure 4).

**Figure 5:**
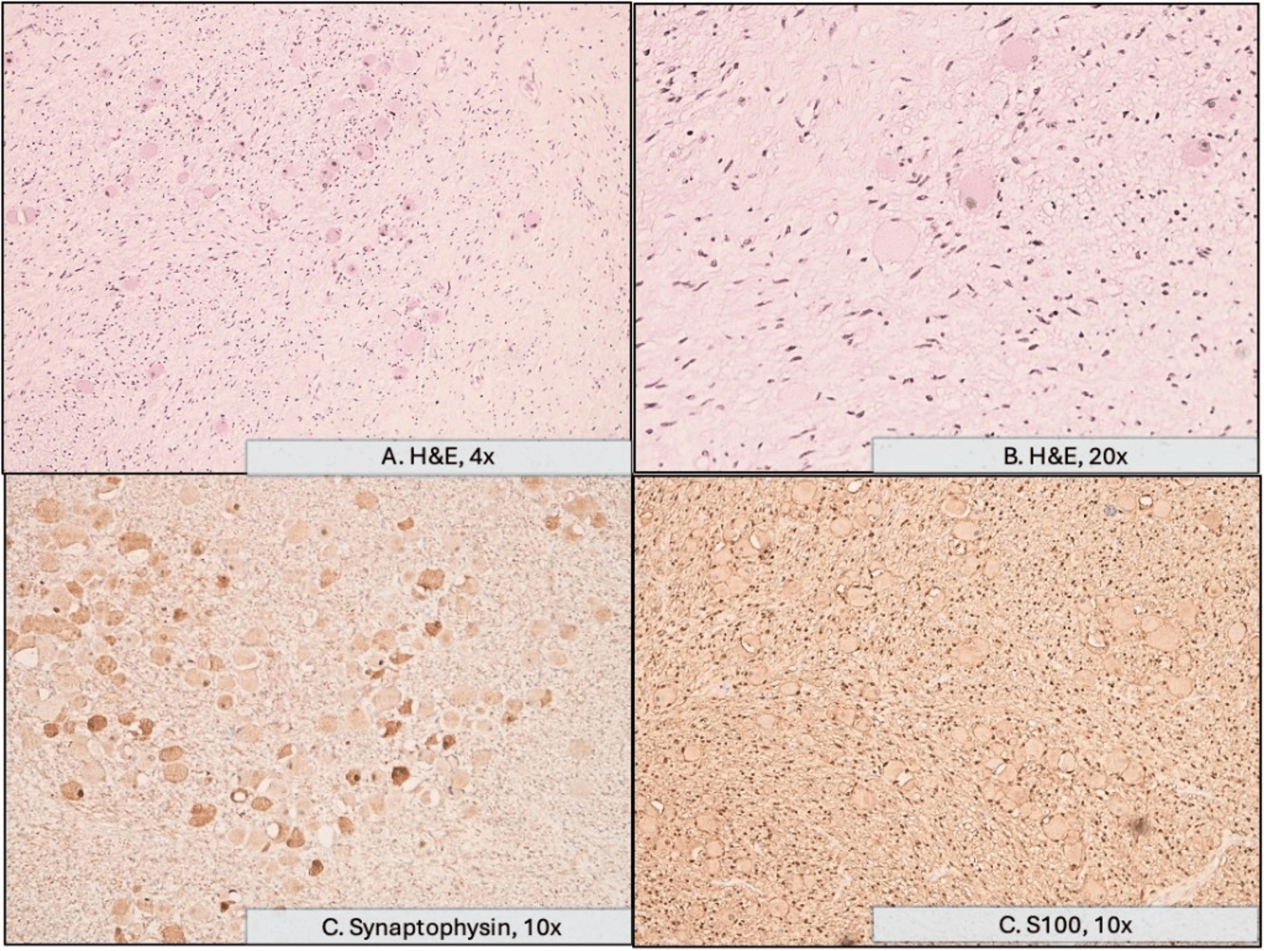
Histopathological and immunohistochemical analysis of the lesion. A: Histological section stained with H&E showing a proliferation composed of Schwann cells and ganglion cells, which are further highlighted in B. C: Ganglion cells exhibit cytoplasmic positivity for synaptophysin, confirming their neuronal differentiation. D: S100 immunostaining highlights the cytoplasmic membranes of the Schwann cells. H&E: hematoxylin and eosin

Bronchoscopy revealed only a slightly widened carina, with otherwise normal right and left bronchial trees. Cytological and microbiological studies of collected secretions were negative. EUS confirmed a large, heterogeneous, hypoechoic posterior mediastinal lesion with anechoic and calcified hyperechoic areas, showing moderate vascularity and appearing extra-esophageal in origin. The mass contoured the thoracic aorta but showed no signs of invasion. Fine-needle aspiration (FNA) was performed without complications, yielding a negative cytological result. The patient subsequently underwent surgical resection of the mass via thoracic surgery. The lesion was completely excised without intraoperative complications. Histopathological examination confirmed the diagnosis of GN of the posterior mediastinum (Figure 5).

**Figure 6:**
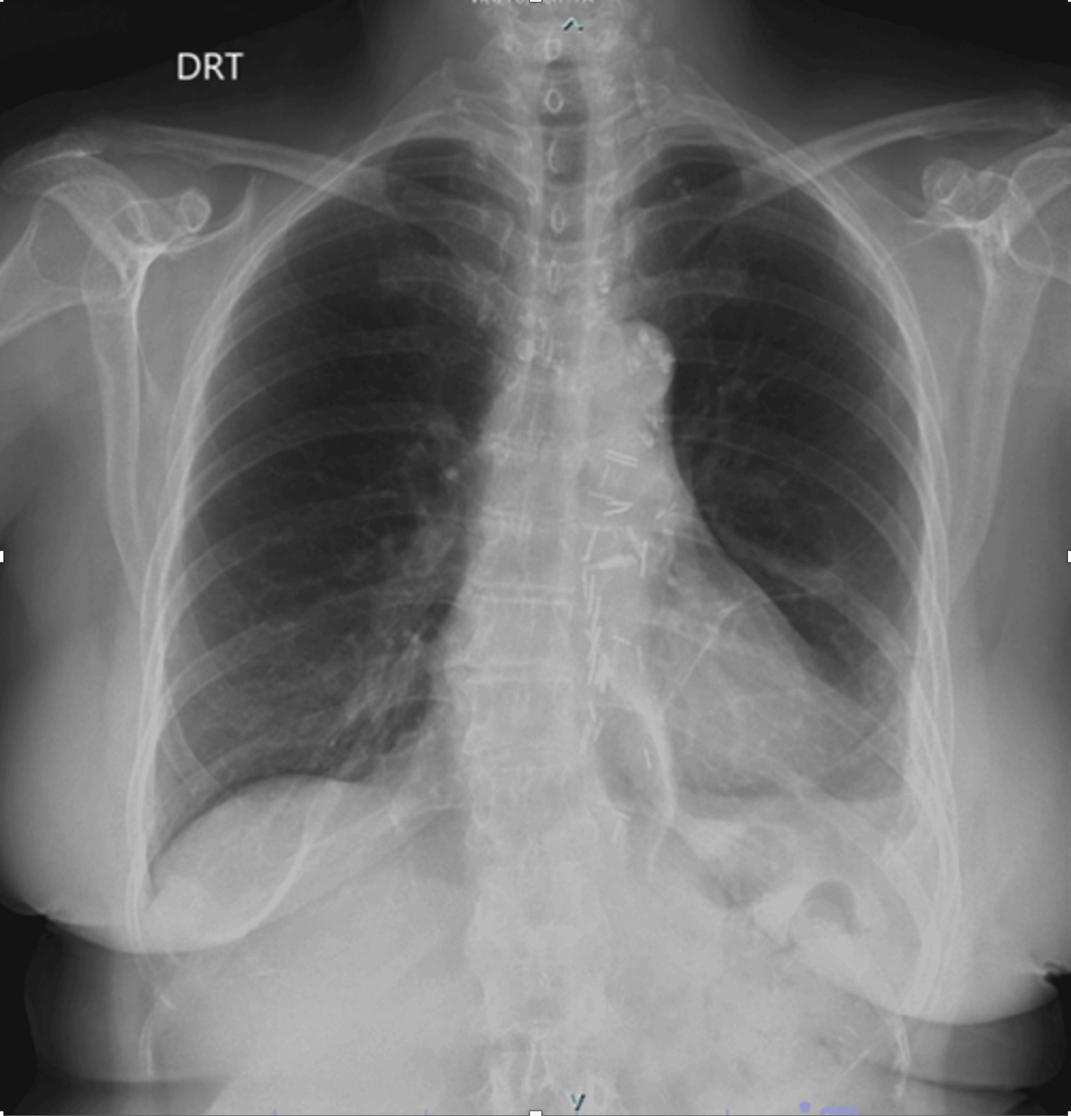
Postoperative chest radiograph performed one month later shows stable postsurgical changes following posterior mediastinal mass resection. Primary findings include a small-to-mild left pleural effusion, with otherwise clear lung fields and no evidence of pneumothorax or significant residual or recurrent disease.

Clinical follow-up demonstrated a clear improvement in the previously reported compressive symptoms. A postoperative reassessment chest radiograph showed no evidence of residual or recurrent posterior mediastinal abnormalities (Figure 6).

## Discussion

GN are rare, benign tumors of neural crest origin, accounting for approximately 0.1%-0.5% of neurological tumors [1]. They most commonly arise in the retroperitoneum and posterior mediastinum [2]. Although they can occur at any age, they have a higher prevalence in young and middle-aged adults [3], making the present case particularly uncommon (while often cited as a tumor of childhood, a systematic review of individual patient data demonstrated that 65.7% of reported GN cases are actually diagnosed in adults [3]). These tumors are typically slow-growing and often asymptomatic, which explains why they are usually discovered at a large size [1]. When voluminous, they may compress adjacent organs and produce symptoms such as discomfort, pain, cough, dyspnea, or chest tightness [4-6]. In this particular case, the tumor’s indolent growth, in conjunction with the thoracic cavity’s ability to adapt to slow expansion, delayed the onset of symptoms. In our patient, the mass remained clinically silent (including a lack of symptoms of catecholamine excess, such as labile hypertension and flushing, as some patients may present with elevated catecholamine levels [2]) until an intercurrent influenza infection prompted further evaluation. This highlights how respiratory infections may unmask previously asymptomatic mediastinal lesions and underscores the importance of considering alternative diagnoses when symptoms persist beyond the expected course of infection. Similar to our patient, whose symptoms (cough and dyspnea) were initially attributed to a post-viral syndrome, other reported adult cases of posterior mediastinal GN are discovered incidentally or during evaluation for nonspecific respiratory complaints [5,6].

Posterior mediastinal lesions are frequently underdiagnosed on standard chest radiographs due to the anatomical overlap of mediastinal structures and the limited contrast resolution in this region [7]. Small or well-circumscribed masses may remain obscured by the cardiac silhouette, vertebral column, or paraspinal musculature, reducing the sensitivity of plain radiography for detecting abnormalities in the posterior compartment [7]. This diagnostic limitation was evident in the present clinical case, in which the mediastinal lesion was not initially identified on chest and abdominal radiographs years before clinical presentation, reinforcing the importance of advanced cross-sectional imaging, such as CT or MRI, when clinical suspicion persists. These imaging studies, which are essential for defining the lesion’s characteristics and anatomical relationships [8], should be a part of the diagnostic approach to suspected posterior mediastinal lesions [8].

In our case, the mass did not invade adjacent organs or tissues, nor did it cause spinal cord compression, despite its large size and proximity to critical mediastinal structures. This finding is consistent with the biological behavior of GN, which are typically well-encapsulated and noninvasive lesions [1-6]. Most reported cases describe similar characteristics, with local compression being the main mechanism of symptom production rather than infiltration of neighboring structures [1-6]. True invasion is exceedingly rare and more often associated with ganglioneuroblastoma or neuroblastoma. In this case, the mass measured about 13x9 cm, which is large but not unprecedented; many reported cases describe tumors between 5 cm and 10 cm, but there are published reports of even larger lesions (up to 20-25 cm, particularly in the retroperitoneum or thoracic cavity) [9]. EUS can provide additional information on vascular or esophageal involvement. It is minimally invasive compared to surgical options such as mediastinoscopy, carries a lower risk of complications, and often avoids thoracotomy. Moreover, EUS can reach lesions in posterior mediastinal locations or near the esophagus that would be difficult or riskier to sample via bronchoscopy or CT-guided needle biopsy [10]. In published studies, EUS-guided FNA/fine-needle biopsy (FNB) for mediastinal lesions has shown high sensitivity, specificity, and accuracy, with few complications [10]. However, FNA by EUS may be nondiagnostic [11], as occurred in this case, reinforcing the role of surgical resection for both therapeutic and diagnostic purposes. Complete surgical excision provides relief of compressive symptoms and rules out malignancy, both observed in this case, and carries an excellent prognosis, as recurrence and malignant transformation are exceedingly rare [1,2,4]. The definitive diagnosis was ultimately established in this case through surgical intervention and anatomopathological analysis of the resected surgical specimen: grossly, the lesion was a well-circumscribed, solid, yellowish mass. Histologically, it showed mature ganglion cells with abundant amphophilic-to-eosinophilic cytoplasm within a Schwannian spindle cell stroma [12,13]. Immunohistochemistry confirmed the neural nature of the lesion, with S100 expression in Schwann cells and synaptophysin positivity in the ganglion cells [12,13].

## Conclusions

GN are rare, benign neurogenic tumors. Although uncommon in older adults, they should be considered as a differential diagnosis, particularly in patients with persistent respiratory compressive symptoms. This case highlights several important aspects of posterior mediastinal GN. First, although the tumor was large, it remained indolent and noninvasive, consistent with the benign behavior of GN. Second, the diagnosis in a 61-year-old patient is unusual, as these tumors are more typically encountered in younger populations. The fact that a concurrent respiratory infection prompted imaging, which incidentally revealed this mass, highlights its slow and silent growth until it reaches a size sufficient to cause compressive symptoms. This emphasizes how posterior mediastinal lesions often go undetected, as their asymptomatic progression and limited visibility on standard chest radiographs contribute to underdiagnosis. This case emphasizes that nonresolving or nonspecific respiratory symptoms, even following a common viral illness, must prompt appropriate imaging in older adults to rule out other causes, including mechanical compression from an underlying mass, as occurred in this case. It also demonstrates the value of a multimodal diagnostic approach, including spectral CT imaging, MRI, bronchoscopy, and EUS-guided biopsy, in establishing the possible nature of the mass and planning surgical management. Still, despite the utility of preoperative imaging modalities, the definitive diagnosis was ultimately established through surgical intervention and analysis of the resected surgical specimen, which in this case confirmed GN (the lesion is characterized by a predominant Schwannian stroma and the presence of fully mature ganglion cells, without evidence of an immature neuroblastic component). Complete resection remains the treatment of choice, ensuring both a definitive diagnosis and an excellent prognosis.
